# AHoJ: rapid, tailored search and retrieval of apo and holo protein structures for user-defined ligands

**DOI:** 10.1093/bioinformatics/btac701

**Published:** 2022-10-25

**Authors:** Christos P Feidakis, Radoslav Krivak, David Hoksza, Marian Novotny

**Affiliations:** Department of Cell Biology, Faculty of Science, Charles University, Prague 12843, Czech Republic; Department of Software Engineering, Faculty of Mathematics and Physics, Charles University, Prague 12116, Czech Republic; Department of Software Engineering, Faculty of Mathematics and Physics, Charles University, Prague 12116, Czech Republic; Department of Cell Biology, Faculty of Science, Charles University, Prague 12843, Czech Republic

## Abstract

**Summary:**

Understanding the mechanism of action of a protein or designing better ligands for it, often requires access to a bound (holo) and an unbound (apo) state of the protein. Resources for the quick and easy retrieval of such conformations are severely limited. Apo–Holo Juxtaposition (AHoJ), is a web application for retrieving apo–holo structure pairs for user-defined ligands. Given a query structure and one or more user-specified ligands, it retrieves all other structures of the same protein that feature the same binding site(s), aligns them, and examines the superimposed binding sites to determine whether each structure is apo or holo, in reference to the query. The resulting superimposed datasets of apo–holo pairs can be visualized and downloaded for further analysis. AHoJ accepts multiple input queries, allowing the creation of customized apo–holo datasets.

**Availability and implementation:**

Freely available for non-commercial use at http://apoholo.cz. Source code available at https://github.com/cusbg/AHoJ-project.

**Supplementary information:**

Supplementary data are available at *Bioinformatics* online.

## 1 Introduction

The study of protein–ligand interactions constitutes a prominent field in structural biology. Observing the effects of ligand binding (Brylinski and Skolnick, 2008), or exploring the specificity of a binding site (Ma *et al.*, 2002), involve studying several protein–ligand interactions. Unveiling cryptic binding sites (Cimermancic *et al.*, 2016), assessing the importance and consistency of water molecules (Wlodawer *et al.*, 2018), or transcending the technical limitations of rigid body docking with ensemble docking methodologies (Amaro *et al.*, 2018), also require access to several conformations (preferably apo and holo).

A number of datasets and tools have been built to address this need. ComSin (Lobanov *et al.*, 2010) comprised a database of apo and holo protein pairs which exhibit significant shifts in their levels of intrinsic disorder upon complex formation. AH-DB (Chang *et al.*, 2012) expanded this scope by including small ligands in its repertoire of apo–holo pairs. The BUDDY-system (Morita *et al.*, 2011) provided a more flexible solution where the user could specify the ligand of interest, and the application would try to pair up the provided holo structure with an apo counterpart. At the time of writing, none of these servers are available. A recent work in preprint (APObind—unpublished data) aims to complement an existing database of protein–ligand complexes, by pairing up the holo complexes with their apo counterparts. LigASite (Dessailly *et al.*, 2008) is a more dated yet surviving resource that features pairs of apo and holo structures for 550 proteins. In both cases however, the ligand cannot be specified by the user.

The available resources appear to be restricted, and in some cases non-existent. The ability to define a ligand, and therefore a binding site, that will guide the search for apo and holo structures is missing altogether. This can be particularly useful as proteins often bind several ligands, and even within the same protein, different structures can bind different ligands in the same or in different binding sites. Therefore, finding pairs of apo and holo structures for a given target structure, requires specifying one or more ligands of interest. A methodology that defines the relevant ligands according to a fixed assumption (i.e. automatically), can restrict a user who wants to focus on a ligand that is deemed irrelevant, or narrow down the search to a single ligand when more bind the same structure. Ultimately, when an application forcefully decides upon the relevance of a ligand, it strips the user of this choice and it is also confronted with the non-trivial matter of biological relevance (Capitani *et al.*, 2016).

Here, we present a web application that enables the user to conduct easy and fast parameterizable searches for apo and holo structure pairs against a target structure, by specifying one or more ligands of interest in this target structure, or letting the application detect the ligands instead. By tracking the binding site of the user-defined ligand across structures, it can construct a repertoire of ligands that bind the same site and enable studies on binding-site specificity.

## 2 Materials and methods

AHoJ starts the search by spatially marking the user-defined ligand(s) and identifying their binding residues with PyMOL. Ligands are typically confined to non-protein chemical moieties, however in AHoJ, the concept of ligand can be extended to include water molecules and modified or non-standard residues (e.g. phosphorylated residues or D-residues) as points of interest or candidate ligands (see Supplementary Information for details).

It then compiles a list of candidate structure chains by (i) detecting the UniProt accession number (AC) (UniProt: the universal protein knowledgebase, 2017) of each query chain and (ii) retrieving all other chains that belong to the same UniProt AC. At the same time, it maps the binding residues of the query ligands onto the UniProt sequence by using the residue-level mappings from SIFTS (Dana *et al.*, 2019), and cross-examines each candidate chain to determine how many of the mapped binding residues are present. If a minimum percentage of binding residues is detected, the chain is considered a successful candidate and it is aligned onto the query chain with TM-align (Zhang and Skolnick, 2005). The user can adjust these parameters (see Supplementary Information for details). The candidate’s area around the superimposed query ligand is examined for ligands, and the results are saved along with the aligned chains. This process is repeated for all candidate chains and each one is listed as holo or apo respective to the presence or absence of ligands in the defined binding site(s). The detected ligands along with metrics for the similarity between candidate and query, presence of binding residues and alignment scores, are reported for each apo and holo chain. The overall workflow is depicted in Supplementary Figure S1. Results are visualized in the browser and can be downloaded locally and loaded into PyMOL through an included script.

## Supplementary Material

btac701_Revised_Supplementary_File
